# Ovarian and Breast Cancer Risks Associated With Pathogenic Variants in *RAD51C* and *RAD51D*

**DOI:** 10.1093/jnci/djaa030

**Published:** 2020-02-28

**Authors:** Xin Yang, Honglin Song, Goska Leslie, Christoph Engel, Eric Hahnen, Bernd Auber, Judit Horváth, Karin Kast, Dieter Niederacher, Clare Turnbull, Richard Houlston, Helen Hanson, Chey Loveday, Jill S Dolinsky, Holly LaDuca, Susan J Ramus, Usha Menon, Adam N Rosenthal, Ian Jacobs, Simon A Gayther, Ed Dicks, Heli Nevanlinna, Kristiina Aittomäki, Liisa M Pelttari, Hans Ehrencrona, Åke Borg, Anders Kvist, Barbara Rivera, Thomas V O Hansen, Malene Djursby, Andrew Lee, Joe Dennis, David D Bowtell, Nadia Traficante, Orland Diez, Judith Balmaña, Stephen B Gruber, Georgia Chenevix-Trench, kConFab Investigators, Allan Jensen, Susanne K Kjær, Estrid Høgdall, Laurent Castéra, Judy Garber, Ramunas Janavicius, Ana Osorio, Lisa Golmard, Ana Vega, Fergus J Couch, Mark Robson, Jacek Gronwald, Susan M Domchek, Julie O Culver, Miguel de la Hoya, Douglas F Easton, William D Foulkes, Marc Tischkowitz, Alfons Meindl, Rita K Schmutzler, Paul D P Pharoah, Antonis C Antoniou

**Affiliations:** d1 Department of Public Health and Primary Care, Centre for Cancer Genetic Epidemiology, University of Cambridge, Cambridge, UK; d2 Department of Oncology, Centre for Cancer Genetic Epidemiology, University of Cambridge, Cambridge, UK; d3 Institute for Medical Informatics, Statistics and Epidemiology, University of Leipzig, Leipzig, Germany; d4 Faculty of Medicine and University Hospital Cologne, Center for Familial Breast and Ovarian Cancer, University of Cologne, Cologne, Germany; d5 Faculty of Medicine and University Hospital Cologne, Center for Integrated Oncology, University of Cologne, Cologne, Germany; d6 Institute of Human Genetics, Hannover Medical School, Hannover, Germany; d7 Institute of Human Genetics, University of Münster, Münster, Germany; d8 Department of Gynecology and Obstetrics, Medical Faculty and University Hospital Carl Gustav Carus, Technische Universität Dresden, Dresden, Germany; d9 National Center for Tumor Diseases (NCT), Dresden, Germany: German Cancer Research Center (DKFZ), Heidelberg, Germany; Faculty of Medicine and University Hospital Carl Gustav Carus, Technische Universität Dresden, Dresden, Germany; Helmholtz-Zentrum Dresden-Rossendorf (HZDR), Dresden, Germany; d10 German Cancer Consortium (DKTK), Dresden and German Cancer Research Center (DKFZ), Heidelberg, Germany; d11 Department of Gynecology and Obstetrics, Heinrich-Heine University Düsseldorf, University Hospital Düsseldorf, Düsseldorf, Germany; d12 Division of Genetics and Epidemiology, The Institute of Cancer Research, London, UK; d13 Ambry Genetics, Aliso Viejo, Canada; d14 School of Women’s and Children’s Health, Faculty of Medicine, University of NSW Sydney, Sydney, New South Wales, Australia; d15 Garvan Institute of Medical Research, The Kinghorn Cancer Centre, Sydney, New South Wales, Australia; d16 Adult Cancer Program, Lowy Cancer Research Centre, University of NSW Sydney, Sydney, New South Wales, Australia; d17 MRC Clinical Trials Unit at UCL, Institute of Clinical Trials and Methodology, University College London, London, UK; d18 Women’s Cancer, Institute for Women’s Health, University College London, London, UK; d19 University of New South Wales, Sydney, New South Wales, Australia; d20 University of Manchester, Manchester, UK; d21 Center for Bioinformatics and Functional Genomics and the Cedars Sinai Genomics Core, Cedars-Sinai Medical Center, Los Angeles, CA, USA; d22 Department of Obstetrics and Gynecology, Helsinki University Hospital, University of Helsinki, Helsinki, Finland; d23 Department of Clinical Genetics, Helsinki University Hospital, University of Helsinki, Helsinki, Finland; d24 Department of Clinical Genetics and Pathology, Laboratory Medicine, Office for Medical Services, Region Skåne, Lund, Sweden; d25 Division of Clinical Genetics, Department of Laboratory Medicine, Lund University, Lund, Sweden; d26 Division of Oncology and Pathology, Department of Clinical Sciences Lund, Lund University, Lund, Sweden; d27 Gerald Bronfman Dept Oncology, Jewish General Hospital, McGill University and Lady Davis Institute, Montréal, QC, Canada; d28 Program in Molecular Mechanisms and Experimental Therapy in Oncology (Oncobell), IDIBELL, Hospitalet de Llobregat, Barcelona, Spain; d29 Center for Genomic Medicine, Rigshospitalet, Copenhagen University Hospital, Copenhagen, Denmark; d30 Department of Clinical Genetics Rigshospitalet, Copenhagen University Hospital, Copenhagen, Denmark; d31 Peter MacCallum Cancer Center, Melbourne, Victoria, Australia; d32 Sir Peter MacCallum, Department of Oncology, The University of Melbourne, Parkville, Victoria, Australia; d33 Oncogenetics Group, Vall dHebron Institute of Oncology, Barcelona, Spain; d34 Clinical and Molecular Genetics Area, University Hospital Vall dHebron, Barcelona, Spain; d35 Hereditary Cancer Genetics Group, Vall d’Hebron Institute of Oncology, Barcelona, Spain; d36 Department of Medical Oncology, University Hospital of Vall d’Hebron, Barcelona, Spain; d37 Department of Preventive Medicine, Keck School of Medicine, University of Southern California, Los Angeles, CA, USA; d38 Department of Genetics and Computational Biology, QIMR Berghofer Medical Research Institute, Brisbane, Queensland, Australia; d39 Kathleen Cuningham Foundation Consortium for research into Familial Breast cancer; d40 Department of Virus, Lifestyle and Genes, Danish Cancer Society Research Center, Copenhagen, Denmark; d41 Department of Gynaecology, University of Copenhagen, Rigshospitalet, Copenhagen, Denmark; d42 Department of Pathology, Molecular Unit, Herlev Hospital, University of Copenhagen, Copenhagen, Denmark; d43 Department of Cancer Biology and Genetics, Normandy Centre for Genomic and Personalized Medicine, François Baclesse Center, Inserm U1245, Caen, France; d44 Cancer Risk and Prevention Clinic, Dana-Farber Cancer Institute, Boston, MA, USA; d45 Department of Molecular and Regenerative Medicine, Hematology, Oncology and Transfusion Medicine Center, Vilnius University Hospital Santariskiu Clinics, Vilnius, Lithuania; d46 State Research Institute Centre for Innovative Medicine, Vilnius, Lithuania; d47 Centro de Investigación en Red de Enfermedades Raras, Madrid, Spain; d48 Human Cancer Genetics Programme, Spanish National Cancer Research Centre, Madrid, Spain; d49 Institut Curie, Paris Sciences Lettres Research University, Service de Génétique, Paris, France; d50 Fundación Pública Galega de Medicina Xenómica, Santiago de Compostela, Spain; d51 Instituto de Investigación Sanitaria de Santiago de Compostela, Complejo Hospitalario Universitario de Santiago, SERGAS, Santiago de Compostela, Spain; d52 Department of Laboratory Medicine and Pathology, Mayo Clinic, Rochester, MN, USA; d53 Department of Medicine, Memorial Sloan Kettering Cancer Center, Clinical Genetics Service, New York, NY, USA; d54 Department of Genetics and Pathology, Pomeranian Medical University, Szczecin, Poland; d55 Basser Center for BRCA, Abramson Cancer Center, University of Pennsylvania, Philadelphia, PA, USA; d56 Keck School of Medicine, University of Southern California, Los Angeles, CA, USA; d57 Molecular Oncology Laboratory CIBERONC, Hospital Clinico San Carlos, IdISSC (Instituto de Investigación Sanitaria del Hospital Clínico San Carlos), Madrid, Spain; d58 Program in Cancer Genetics, Departments of Human Genetics and Oncology, McGill University, Montréal, QC, Canada; d59 Department of Medical Genetics, National Institute for Health Research Cambridge Biomedical Research Centre, University of Cambridge, Cambridge, UK; d60 Department of Gynecology and Obstetrics, University of Munich, Campus Großhadern, Munich, Germany; d61 Faculty of Medicine and University Hospital Cologne, Center for Molecular Medicine Cologne, University of Cologne, Cologne, Germany

## Abstract

**Background:**

The purpose of this study was to estimate precise age-specific tubo-ovarian carcinoma (TOC) and breast cancer (BC) risks for carriers of pathogenic variants in *RAD51C* and *RAD51D*.

**Methods:**

We analyzed data from 6178 families, 125 with pathogenic variants in *RAD51C*, and 6690 families, 60 with pathogenic variants in *RAD51D.* TOC and BC relative and cumulative risks were estimated using complex segregation analysis to model the cancer inheritance patterns in families while adjusting for the mode of ascertainment of each family. All statistical tests were two-sided.

**Results:**

Pathogenic variants in both *RAD51C* and *RAD51D* were associated with TOC (*RAD51C*: relative risk [RR] = 7.55, 95% confidence interval [CI] = 5.60 to 10.19; *P* = 5 × 10^-40^; *RAD51D*: RR = 7.60, 95% CI = 5.61 to 10.30; *P* = 5 × 10^-39^) and BC (*RAD51C*: RR = 1.99, 95% CI = 1.39 to 2.85; *P* = 1.55 × 10^-4^; *RAD51D*: RR = 1.83, 95% CI = 1.24 to 2.72; *P* = .002). For both *RAD51C* and *RAD51D*, there was a suggestion that the TOC relative risks increased with age until around age 60 years and decreased thereafter. The estimated cumulative risks of developing TOC to age 80 years were 11% (95% CI = 6% to 21%) for *RAD51C* and 13% (95% CI = 7% to 23%) for *RAD51D* pathogenic variant carriers. The estimated cumulative risks of developing BC to 80 years were 21% (95% CI = 15% to 29%) for *RAD51C* and 20% (95% CI = 14% to 28%) for *RAD51D* pathogenic variant carriers. Both TOC and BC risks for *RAD51C* and *RAD51D* pathogenic variant carriers varied by cancer family history and could be as high as 32–36% for TOC, for carriers with two first-degree relatives diagnosed with TOC, or 44–46% for BC, for carriers with two first-degree relatives diagnosed with BC.

**Conclusions:**

These estimates will facilitate the genetic counseling of *RAD51C* and *RAD51D* pathogenic variant carriers and justify the incorporation of *RAD51C* and *RAD51D* into cancer risk prediction models.

Genetic testing through multigene cancer panels is widely available and has become an integral part of the genetic counseling and oncologic practice used to inform clinical management options. *RAD51C* and *RAD51D* are included on widely available cancer panels because of the reported associations of pathogenic variants in these genes with tubo-ovarian carcinoma (TOC) (1–3). However, the optimal interpretation of gene-panel testing results requires precise cancer risk estimates for pathogenic variants in *RAD51C*.

The reported TOC risks for *RAD51C* pathogenic variant carriers vary widely with odds ratio (OR) estimates ranging from 3.4 to 15.8 based on case-control studies and a relative risk (RR) of 5.9 using family-based segregation analysis (Supplementary Table 1, available online). Similarly, the reported TOC odds ratios and relative risks for *RAD51D* pathogenic variant carriers ranged from 6.3 to 12.0 (Supplementary Table 1, available online). There has been conflicting evidence for the association of both *RAD51C* and *RAD51D* pathogenic variants with breast cancer (BC) risk. Some studies reported an increased BC risk (OR estimates for *RAD51C* = 5.9–8.7; *RAD51D* = 3.1–8.3), but others reported no statistically significant associations (Supplementary Table 2, available online) (4–6).

A concern with published risk estimates based on case-control studies has been that cases may have been selected on the basis of cancer family history, which may confound the associations and/or lead to an overestimation of cancer risks because of the enrichment of cases for pathogenic variants. Furthermore, the pathogenic variant frequencies in controls come predominantly from publicly available resources and may come from populations that do not closely match the case population. Therefore, some of the published risk estimates may be susceptible to selection biases or biases because of population stratification and cannot be readily applied in the counseling process. Family- or pedigree-based approaches, with appropriate ascertainment corrections in the analysis, which adjust for the ascertainment process of each family, address directly such potential biases and can result in more precise risk estimates because of the use of information on both genotyped and nongenotyped family members. Here, we use a large collection of families with *RAD51C* and/or *RAD51D* pathogenic variants to estimate age-specific TOC and BC risks and assess how these vary by family history of cancer.

## Methods

### Families

Families were enrolled between 1996 and 2017 through 28 study centers from 12 countries from Europe and North America and were ascertained through *RAD51C* or *RAD51D* variant screening of families with multiple TOC- or BC-affected members (24 studies) and *RAD51C* or *RAD51D* variant screening of TOC or BC patients unselected for cancer family history (three studies). One study included families ascertained through both schemes. Four studies provided data on all families screened for *RAD51C* or *RAD51D* variants, irrespective of the result (Supplementary Table 3, available online). Participants provided informed consent in accordance with institutional review board policies and local practices. The list of study centers and ascertainment criteria are provided in Supplementary Table 3 (available online).

### Variants

Pathogenic variants including frameshift, nonsense, canonical splice sites, and large genomic deletions were considered in the analyses. Variants in the last exon were excluded. We estimated the population *RAD51C* and *RAD51D* variant using the UK Biobank exome sequencing dataset (http://www.ukbiobank.ac.uk).

### Statistical Analysis

Cancer inheritance patterns and observed genotypes in families were modeled using complex segregation analysis to estimate TOC and BC relative risks simultaneously (7, 8) in the pedigree analysis software Mendel, version 3.3 (9).

Family members were followed from birth until the age at first cancer diagnosis (excluding nonmelanoma skin cancer), age at death, age at last follow-up, age at risk-reducing surgery (bilateral mastectomy in the BC analyses or bilateral salpingo-oophorectomy in the TOC analyses if they occurred at least 1 year prior to cancer diagnosis), or age 80 years, whichever occurred first. Women diagnosed with a first TOC or BC were assumed to be affected at the age of diagnosis, whereas women with any other type of first cancer diagnosis were censored at the age of diagnosis and were assumed as unaffected. Missing ages were inferred from other information (Supplementary Methods, available online). Individuals with unknown disease status and no age information were censored at age 0 years.

Each female was assumed to be at risk of developing TOC and BC assuming that the probability of developing TOC was independent of the probability of developing BC conditional on genotype. We modeled the TOC and BC incidences so that they depend on the underlying assumed genetic effects (Supplementary Methods, available online). Two main genetic models were fitted: a major-gene model that assumed all familial aggregation of TOC and BC was explained by *RAD51C* or *RAD51D* and a major-gene plus polygenic component model that considered an additional residual familial component representing other unobserved genetic effects not due to *RAD51C* or *RAD51D* (10,11) (Supplementary Methods, available online). Models were fitted in which the log-relative risk for *RAD51C* or* RAD51D* pathogenic variant carriers relative to population incidences were assumed to be either constant across the whole age range, constant for specific age groups, or a piecewise linear function of age (Supplementary Methods, available online). We used country, cohort, and population age-specific incidences and constrained the overall cancer age-specific incidences over all assumed genetic effects to agree with the population age-specific incidences (11,12) (Supplementary Methods, available online).

Because families were ascertained through different criteria across studies, we employed the ascertainment assumption-free approach to adjust for ascertainment by computing the pedigree likelihood conditional on all data relevant to the ascertainment (13–15) (Supplementary Methods, available online). Noninformative families, for which no additional information was available beyond the data relevant to the ascertainment, were excluded from the analysis.

The most parsimonious models were selected by either comparing the Akaike information criterion (AIC) for nonnested models, by selecting the model with the smaller AIC, or using the likelihood ratio test (LRT) for nested models. The hypothesis that the relative risk is 1.00 was assessed using a Wald test statistic. All statistical tests were two-sided. Statistical significance was considered as a *P* value less than 0.05.

## Results

### Variants and Families

A total of 7216 families eligible for pathogenic variant analysis were submitted to the coordinating center, where 6049 were identified through individuals with multiple relatives diagnosed with TOC or BC, and 1167 were identified through women diagnosed with TOC or BC unselected for cancer family history. After adjustment for ascertainment, 6178 and 6690 families were eligible for the *RAD51C* and *RAD51D* penetrance analysis, respectively (Supplementary Tables 3 and 4, available online). These included 215 women with *RAD51C* pathogenic variants (137 were TOC or BC cases) from 125 families and 92 women with *RAD51D* pathogenic variants (66 were TOC or BC cases) from 60 families (Table 1). Full lists of the *RAD51C* and *RAD51D* pathogenic variants in this dataset are summarized in Supplementary Tables 5 and 6 (available online). The pathogenic variant population frequencies used in the segregation analysis model were estimated to be 0.00022 for *RAD51C* and 0.00026 for *RAD51D* based on 42 325 cancer-free individuals from the UK Biobank exome sequencing data.

**Table 1.** Summary of women by mutation, disease status, and age among the families with *RAD51C* and *RAD51D* pathogenic variants

Age, y	Pathogenic variant carriers			Tested noncarriers			Untested		
	Unaffected	BC	TOC	Unaffected	BC	TOC	Unaffected	BC	TOC
*RAD51C* (n = 1794 from 125 families)									
<20	1	0	0	1	0	0	88	0	1
20–29	6	1	0	2	0	0	73	4	1
30–39	18	21	2	12	1	0	128	15	6
40–49	26	25	10	24	4	0	156	35	12
50–59	14	16	27	11	3	0	143	30	21
60–69	9	6	20	9	5	2	161	35	24
70–80	4	4	6	3	1	0	368	15	15
Missing*	0	0	0	0	0	0	172	0	0
Total†	78	73	65	62	14	2	1289	134	80
*RAD51D* (n = 935 from 60 families)									
<20	1	0	0	2	0	0	26	0	0
20–29	2	1	0	2	0	0	40	0	0
30–39	7	7	2	6	0	0	54	7	4
40–49	7	11	4	8	2	1	80	19	7
50–59	7	8	17	8	0	0	85	28	19
60–69	1	3	10	5	2	0	87	13	14
70–80	1	0	3	0	0	0	192	7	5
Missing*	0	0	0	0	0	0	120	0	0
Total	26	30	36	31	4	1	684	74	49

* Individuals with missing phenotype information were censored at age 0 years. BC = breast cancer; TOC = tubo-ovarian carcinoma.

† There are three individuals with two cancers diagnosed at the same age and counted in both BC and TOC: one is *RAD51C* pathogenic variant carrier, and the other two were untested for *RAD51C*.

### Risk Models

The genetic models that included a residual polygenic component for TOC and BC provided better fits to the data than the major-gene models for both *RAD51C* and *RAD51D* (results for major gene models not shown). For *RAD51C,* using a constant relative risk with age, the AIC for the major gene model was 4363 compared with 4346 for the BC polygenic model and with 4336 for the TOC polygenic model (Table 2). For *RAD51D,* the AIC for the major-gene model was 4187 compared with 4178 for the BC polygenic model and with 4160 for the TOC polygenic model (Table 2). Therefore, we based all subsequent analyses on the major-gene plus polygenic component models.

**Table 2.** Estimated tubo-ovarian carcinoma and breast cancer relative risk for *RAD51C* and *RAD51D* pathogenic variant carriers

Cancer and models considered	Age, y	RR (95% CI)	*P* *	LRT *P*†	AIC	Best fitting models
*RAD51C*						
Tubo-ovarian carcinoma						
Age-constant model	30–79	7.55 (5.60 to 10.19)	5 × 10^-40^		4335.8	
Age-specific model for each decade of age	30–39	2.85 (0.46 to 17.70)		0.04	4334.0	
	40–49	5.94 (3.09 to 11.43)				
	50–59	8.55 (5.10 to 14.33)				
	60–69	13.90 (8.45 to 22.88)				
	70–79	2.54 (0.53 to 12.27)				
Age-specific model, separate parameters for two age groups: 30–50 and 50–80 y	30–49	4.97 (2.75 to 8.97)		0.048	4333.8	
	50–79	9.44 (6.63 to 13.45)				
Piecewise linear model‡	35	2.40		0.004	4328.6	Yes
	45	5.14				
	55	11.02				
	65	9.02				
	75	2.82				
Breast cancer						
Age-constant model	20–79	1.99 (1.39 to 2.85)	1.55 × 10^-4^		4346.4	Yes
Age-specific model, separate parameters for each decade of age	20–29	1.19 (0.09 to 16.12)		0.37	4351.0	
	30–39	3.25 (1.60 to 6.62)				
	40–49	2.50 (1.41 to 4.45)				
	50–59	0.96 (0.34 to 2.71)				
	60–69	1.54 (0.45 to 5.36)				
	70–79	2.57 (0.61 to 10.81)				
Age-specific model, separate parameters for two age groups: 20–50 and 50–80 y	20–49	2.42 (1.61 to 3.63)		0.12	4346.0	
	50–79	1.36 (0.70 to 2.63)				
*RAD51D*						
Tubo-ovarian carcinoma						
Age-constant model	30–79	7.60 (5.61 to 10.30)	5 × 10^-39^		4160.0	
Age-specific model for each decade of age	30–39	3.60 (0.78 to 16.75)		0.02	4155.8	
	40–49	3.19 (1.04 to 9.72)				
	50–59	12.54 (7.62 to 20.63)	—			
	60–69	10.60 (6.10 to 18.41)	—			
	70–79	4.94 (1.34 to 18.26)	—			
Age-specific model, separate parameters for two age groups: 30–50 and 50–80 y	30–49	3.23 (1.36 to 7.71)	—	0.002	4152.1	
	50–79	10.56 (7.48 to 14.91)	—			
Piecewise linear model§	35	1.64	—	0.002	4151.6	Yes
	45	4.30	—			
	55	11.29	—			
	65	10.16	—			
	75	5.77	—			
Breast cancer						
Age-constant model	20–79	1.83 (1.24 to 2.72)	0.0002		4177.9	Yes
Age-specific model, separate parameters for each decade of age except for 20–39 y age group	20–39	2.25 (1.25 to 4.04)	—	0.59	4183.1	
	40–49	1.46 (0.69 to 3.09)	—			
	50–59	1.56 (0.69 to 3.51)	—			
	60–69	1.63 (0.54 to 4.98)	—			
	70–79	4.19 (1.51 to 11.62)	—			
Age-specific model, separate parameters for two age groups: 20–50 and 50–80 y	20–49	1.84 (1.12 to 3.02)	—	1.00	4179.9	
	50–79	1.83 (1.02 to 3.26)	—			

* The *P* values assessing the null hypothesis of RR = 1.00. AIC = Akaike information criterion; CI = confidence interval; RR = relative risk.

† Likelihood ratio tests (LRT) comparing each model against the model with a constant RR.

‡ logRR(t) = a + b_1_(t − 30) if t ∈ [30,60); logRR(t) = a + b_1_ × 30 + b_2_(t − 60) if t ∈ [60,80), where a = 0.49 (95% CI = −0.80 to 1.78), b_1_ = 0.076 (95% CI = 0.023 to 0.13), b_2_ = −0.12 (95% CI = −0.23 to −0.0036).

§ logRR(t) = a + b_1_(t − 30) if t ∈ [30,58); logRR(t) = a + b_1_ × 28 + b_2_(t − 58) if t ∈ [58,80), where a = 0.010 (95% CI = −1.49 to 1.51), b_1_ = 0.097 (95% CI = 0.034 to 0.16), b_2_ = −0.057 (95% CI = −0.13 to 0.017).

### TOC Risk

**Figure 1.**
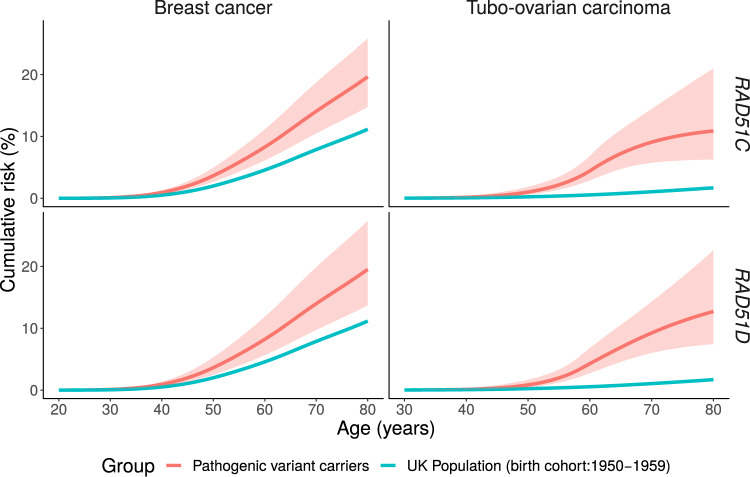
Estimated age-specific tubo-ovarian carcinoma and breast cancer cumulative risks in *RAD51C* and *RAD51D* pathogenic variant carriers. The shaded areas correspond to the 95% confidence intervals.

The estimated TOC relative risks were 7.55 (95% CI = 5.60 to 10.19; *P* = 5 × 10^-^^40^) for *RAD51C* and 7.60 (95% CI = 5.61 to 10.30; *P* = 5 × 10^-^^39^) for *RAD51D* pathogenic variant carriers when relative risks were assumed to be constant with age (Table 2). When separate relative risks were estimated for each age-decade, there was a suggestion that relative risks increased with age until 60–69 years and then decreased for *RAD51C* pathogenic variant carriers. A similar pattern was seen for *RAD51D* pathogenic variant carriers but the relative risk peaked in the 50–59 years age group (Table 2). These models provided a better fit to the data than the models with a constant RR for both *RAD51C* (LRT, degrees of freedom [*df*] = 4; *P* = .04) and *RAD51D* (LRT, *df*  = 4; *P* = .02). When we partitioned age into younger than 50 years and 50 years and older, the estimated TOC relative risks were higher for ages 50 years and older for both *RAD51C* (RR = 9.44, 95% CI = 6.63 to 13.45 for ages  50 years and older; RR = 4.97, 95% CI = 2.75 to 8.97 for ages younger than 50 years) and *RAD51D* pathogenic variant carriers (RR = 10.56, 95% CI = 7.48 to 14.91 for ages 50 years and older; RR = 3.23, 95% CI = 1.36 to 7.71 for ages younger than 50 years). The model with separate relative risk parameters for each decade of age did not fit better than this two age-group model in either *RAD51C* (LRT, *df* = 3; *P* = .12) or *RAD51D* (LRT, *df* = 3; *P* = .51). To smooth the relative risk changes over age, we fitted models in which the log-relative risk was assumed to be a piecewise linear function of age. For *RAD51C*, there was statistically significant evidence that the relative risk increases with age (*P* = .004) from age 30 to 60 years and then decreases. Similarly for *RAD51D*, there was statistically significant evidence that the relative risk increases with age (*P* = .002) from age 30 to 58 years and then decreases. The piecewise linear models were the most parsimonious with the lowest AIC (Table 2). Under these models, the estimated cumulative risks of developing TOC for a woman with a *RAD51C* pathogenic variant to age 50 years was 1% (95% CI = 0.6% to 2%) and 11% (95% CI = 6% to 21%) to age 80 years*;* the corresponding cumulative TOC risks were 0.8% (95% CI = 0.5% to 2%) to age 50 years and 13% (95% CI = 7% to 23%) to age 80 years for a woman with a *RAD51D* pathogenic variant, assuming the UK incidences (Figure 1 and Table 3). The corresponding risks using US population incidences are shown in Supplementary Table 7 (available online).

**Table 3.** Estimated age-specific cancer incidences and cumulative cancer risks for *RAD51C* and *RAD51D* pathogenic variant carriers

Age, y	*RAD51C* pathogenic variant carriers		*RAD51D* pathogenic variant carriers	
	BC	TOC	BC	TOC
Estimated incidences per 1000 person-years (95% CI)*				
30	0.4 (0.2 to 0.5)	0.05 (0.01 to 0.2)	0.3 (0.2 to 0.5)	0.03 (0.007 to 0.1)
40	2 (1 to 3)	0.3 (0.2 to 0.8)	2 (1 to 2)	0.3 (0.1 to 0.7)
50	5 (3 to 6)	2 (1 to 3)	4 (3 to 6)	2 (1 to 3)
60	6 (4 to 9)	7 (4 to 11)	6 (4 to 9)	6 (4 to 8)
70	7 (5 to 10)	3 (1 to 8)	7 (4 to 10)	5 (2 to 9)
79	8 (5 to 11)	1 (0.2 to 8)	7 (5 to 11)	3 (0.9 to 12)
Estimated cumulative risks, % (95% CI)*				
30	0.1 (0.08 to 0.2)	0.02 (0.02 to 0.02)	0.1 (0.07 to 0.2)	0.02 (0.02 to 0.02)
40	1 (0.7 to 1)	0.2 (0.08 to 0.4)	0.9 (0.6 to 1)	0.1 (0.06 to 0.3)
50	4 (3 to 6)	1 (0.6 to 2)	4 (2 to 5)	0.8 (0.5 to 2)
60	9 (6 to 12)	4 (3 to 7)	8 (6 to 12)	4 (3 to 7)
70	15 (11 to 21)	9 (6 to 14)	14 (10 to 20)	9 (6 to 14)
80	21 (15 to 29)	11 (6 to 21)	20 (14 to 28)	13 (7 to 23)

* Assuming the UK population calendar and cohort-specific incidences for an individual born between 1950 and 1959. Mortality is not accounted for absolute risk estimates. BC = breast cancer; CI = confidence interval; TOC = tubo-ovarian carcinoma.

### Breast Cancer Risk

The estimated BC relative risk was 1.99 (95% CI = 1.39 to 2.85; *P* = 1.55 × 10^-^^4^) for *RAD51C* and 1.83 (95% CI = 1.24 to 2.72; *P* = .002) for *RAD51D* pathogenic variant carriers when relative risk was constant with age (Table 2). When relative risks varied by age-decade, for *RAD51C*, the statistically significant association was restricted to ages 30–49 years, but this model did not fit better than the model with a constant relative risk (LRT, *df* = 5; *P* = .37). When only two age groups were assumed, there was further evidence of higher BC relative risk in younger ages (20–49 years: RR = 2.42, 95% CI = 1.61 to 3.63) compared with ages 50 years and older (RR = 1.36, 95% CI = 0.70 to 2.63), but the model with a constant relative risk remained the most parsimonious. For *RAD51D*, a U-shape pattern was observed with higher relative risk estimates in ages 20–39 and 70–79 years (Table 2), but the model with constant relative risk was the most parsimonious (LRT, *df* = 4; *P* = .59, comparing against the age-specific RR model; Table 2). The estimated cumulative risks of developing BC to age 50 years were 4% (95% CI = 3% to 6%) for *RAD51C* and 4% (95% CI = 2% to 5%) for *RAD51D* pathogenic variant carriers and to age 80 years were 21% (95% CI = 15% to 29%) for *RAD51C* and 20% (95 CI = 14% to 28%) for *RAD51D* pathogenic variant carriers assuming UK incidences (Figure 1 and Table 3; Supplementary Table 7, available online, assuming US incidences).

### Birth Cohort and Variant Screening Sensitivity

We assessed whether the estimated risks vary by birth cohort by estimating separate relative risks for different birth cohort groupings (Supplementary Table 8, available online). There was a suggestion of increasing BC risks with more recent birth cohort, but the differences were not statistically significant. Similarly, there were no statistically significant differences in the TOC relative risk estimates between cohort groupings for either *RAD51C* or *RAD51D* relative risks. We also assessed the impact on the results of assuming a reduced mutation screening sensitivity when including *RAD51C/D* test-negative families (Supplementary Methods, available online). As the mutation screening sensitivity parameter decreased, the estimated TOC and BC relative risks increased (Supplementary Table 9, available online).

### Predicted Risks by Family History

**Figure 2.**
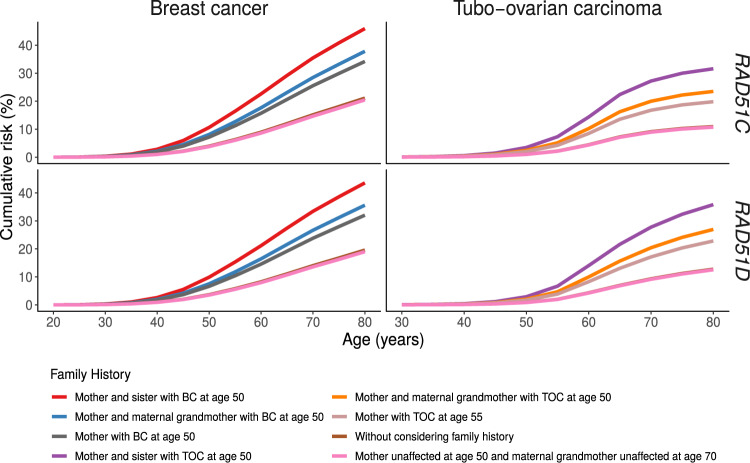
Estimated TOC and BC cumulative risks for *RAD51C* and *RAD51D* pathogenic variant carriers by cancer family history. BC = breast cancer; TOC = tubo-ovarian carcinoma.

The most parsimonious models included a residual familial polygenic component. Under this model, the risk of developing TOC or BC for *RAD51C/D* pathogenic variant differs by cancer family history. The predicted risk of developing TOC to age 80 years varies from 11% (95% CI = 6% to 21%) for *RAD51C* and 13% (95% CI = 7% to 23%) for *RAD51D* pathogenic variant carriers with no family history of TOC in first- and second-degree relatives to 32% (95% CI = 20% to 50%) for *RAD51C* and 36% (95% CI = 23% to 53%) for *RAD51D* pathogenic variant carriers whose mother and sister developed TOC at age 50 years (Figure 2;  Supplementary Tables 10 and 11, available online). Similarly, the predicted cumulative risk of developing BC to age 80 years varies from 20% (95% CI = 15% to 28%) for *RAD51C* and 19% (95% CI = 13% to 27%) for *RAD51D* pathogenic variant carriers with an unaffected mother at age 50 years and unaffected maternal grandmother at age 70 years to 46% (95% CI = 6% to 56%) for *RAD51C* and 44% (95% CI = 33% to 55%) for *RAD51D* pathogenic variant carriers with two first-degree relatives diagnosed with BC (Figure 2;  Supplementary Tables 10 and 11, available online).

## Discussion

This is the largest family-based study to date to estimate age-specific relative and absolute TOC and BC risks for *RAD51C* and *RAD51D* pathogenic variant carriers, confirming that *RAD51C* and *RAD51D* pathogenic variants are associated with TOC and BC risks, which vary by cancer-family history.

Several case-control studies have estimated the association between *RAD51C* and *RAD51D* pathogenic variants and TOC (Supplementary Table 1, available online). However, these studies had limited statistical power and the odds ratio estimates, ranging from 3.4 to 15.8, were imprecise with broad confidence intervals (Supplementary Table 1, available online). The reported associations with BC risk have been unclear with conflicting evidence (Supplementary Table 2, available online). A complicating factor in interpreting the results from some BC case-control studies includes the fact that BC cases may have been selected on the basis of family history of both BC and TOC, which may confound the BC associations given the known TOC association, and publicly available controls were often not closely matched to the case populations. In contrast, the present analysis considered the ascertainment process for each family separately and modeled the simultaneous associations with TOC and BC. In addition, family-based analyses closely control for population stratification because genetic background is shared within families (16, 17).

For both *RAD51C* and *RAD51D* pathogenic variants, the TOC incidence markedly increases and peaks around ages 58–60 years compared with the country- and cohort-specific population incidences. Even though this is the largest study to date, the age-specific results were based on relatively small numbers in each age group. If this pattern is replicated by other studies, it may have implications on the timing of risk-reducing interventions.

We used variant frequencies estimated from the United Kingdom (*RAD51C*: 0.00022; *RAD51D*: 0.00026). These are similar to other frequency estimates. Following the same pathogenic variant selection criteria, the variant frequencies were estimated to be 0.00055 for *RAD51C* and 0.0003 for *RAD51D* using European non-Finnish noncancer gnomAD data and 0.0007 for *RAD51C* and 0.0004 for *RAD51D* from Song et al. (18). Therefore, our results are unlikely to have been influenced by incorrect assumptions for the population variant frequencies.

To maximize the number of families used in the analyses, for studies with data available for all families used in the mutation screening process, we used both families in which pathogenic variants were detected and families without pathogenic variants, under the assumption that the mutation screening sensitivity is 100%. Our analyses, which assumed reduced mutation screening sensitivity, suggest that if this is substantially lower (approximately 60%), the estimated risks may have been somewhat underestimated. The results were very similar to the main results for the most plausible values of 80–90%.

Women diagnosed with cancer were censored at the age of risk-reducing surgery if the surgery occurred at least 1 year prior to cancer diagnosis. We repeated the analysis assuming women were censored at the age of risk-reducing surgery plus 1 year for both affected and unaffected. The results were almost identical to the main analysis (Supplementary Table 12, available online) suggesting that this assumption is unlikely to have led to bias in the results because of unequal counting of person-time.

The most parsimonious models incorporated a residual polygenic component, which also modifies the TOC and BC risk for pathogenic variant carriers. This indicates that other unobserved genetic or environmental risk factors shared in families may modify cancer risks for pathogenic variant carriers, consistent with results on other susceptibility genes [eg, *BRCA1*, *BRCA2*, *PALB2* and *CHEK2* (10, 11, 19–23)]. These may include the combined effects of common genetic variants (polygenic risk score) identified through genome-wide association studies, which have been shown to modify TOC and BC risks for pathogenic variant carriers in other genes (24, 25). The results presented here imply that cancer family history should be considered when counseling carriers with *RAD51C* or* RAD51D* pathogenic variants because it can lead to large differences in the cumulative TOC and BC and thus influence clinical management. For example, the cumulative risk of TOC to age 80 years could be as high as 20–23% for a woman with a *RAD51C* or* RAD51D* pathogenic variant if her mother developed TOC at age 55 years (Figure 2;  Supplementary Tables 10 and 11, available online). Similarly, a woman with a *RAD51C* or *RAD51D* pathogenic variant and a first-degree relative diagnosed with BC at a young age would be classified as high risk (≥30%) of developing BC on the basis of the current National Institute for Health and Care Excellence guidelines in the United Kingdom (26).

The current study has several limitations. Although this is the largest study of its kind to date, we were not able to assess variations in risks by variant type or location. Similarly, the number of TOC and BC cases in some age groups remains small, and age-specific relative risk estimates are associated with large standard errors (Table 2). Previous studies have suggested that pathogenic variants in *RAD51C* or *RAD51D* may be more strongly associated with specific BC subtypes, in particular estrogen receptor–negative or triple-negative BC (5, 6). No cancer subtype analyses were performed for either BC or TOC. To estimate subtype-specific associations in this study design requires tumor pathology data being available on all family members diagnosed with BC or TOC, but these were not available. Nevertheless, our BC risk estimates will still be of clinical relevance as current screening or other interventions do not distinguish between the risks for different BC subtypes. The analysis was restricted to studies from Europe and North America. Further studies are needed when applying our findings to other populations.

It has been recently suggested that risk-reducing salpingo-oophorectomy may be offered to women with more than 4–5% lifetime risk of TOC (27, 28). The current cumulative risk estimates and associated confidence intervals place both *RAD51C* and *RAD51D* pathogenic variant carriers in the category of women for whom risk-reducing salpingo-oophorectomy could be recommended for prevention. However, unlike *BRCA1* pathogenic variants, this may be warranted only for women older than 50 years, which allows for women of childbearing age to complete their families. Although the average risk estimates of BC for *RAD51C/RAD51D* pathogenic variant carriers would place these women in the moderate risk category, in combination with family history of BC, the cumulative risks could be as high as 46% (Figure 2), which would place them in the high-risk category based on the National Institute for Health and Care Excellence guidelines (26).

In summary, we refined and provided age-specific TOC risk estimates for women with *RAD51C* and *RAD51D* pathogenic variants. We also confirmed that both *RAD51C* and *RAD51D* pathogenic variants confer a moderate risk of BC. Our results suggest that the *RAD51C* and *RAD51D* genes should be included in gene panel testing for TOC and BC to guide cancer surveillance and prevention. Incorporation of *RAD51C* and *RAD51D* into risk prediction models should be considered to facilitate stratified TOC and BC risk management.

## Funding

This work was supported by Cancer Research UK (C12292/A20861). ANR and UM were supported by the NIHR Biomedical Research Centre at University College London Hospitals National Health Service Foundation Trust and University College London. BR is supported by a Cancer Research Society grant (OG-24377). JB was supported by the Carlos III National Health Institute funded by FEDER funds – a way to build Europe (PI16/11363). AO has received funding from the Spanish Instituto de Salud Carlos III (PI19/00640) supported by FEDER funds and Centro de Investigación en Red de Enfermedades Raras. AV is supported by the Spanish Health Research Foundation, Instituto de Salud Carlos III, partially supported by FEDER funds through Research Activity Intensification Program (INT15/00070, INT16/00154, INT17/00133) and through Centro de Investigación Biomédica en Red de Enferemdades Raras CIBERER (ACCI 2016: ER17P1AC7112/2018), Autonomous Government of Galicia (Consolidation and structuring program: IN607B), and by the Fundación Mutua Madrileña (call 2018). MH has received funding from the European Union’s Horizon 2020 research and innovation program (634935) and from Spanish Instituto de Salud Carlos III (PI15/00059), an initiative of the Spanish Ministry of economy and innovation partially supported by European regional development Feder Funds. WDF was funded by a Canadian Institutes of Health Research Foundation Grant (FDN-148390). UM receives support from MRC core funding (MR_UU_12023). MT is funded by the European Union Seventh Framework Program (2007e2013)/European Research Council (310018) and by the NIHR Cambridge Biomedical Research Centre. UKFOCSS study data collection and sequencing was funded by the Eve Appeal and Cancer Research UK (C1005/A12677). Funding for MALOVA was provided by a research grant from the National Cancer Institute, Bethesda, MD (R01-CA61107); from the Danish Cancer Society, Copenhagen, Denmark (94 222 52); and the Mermaid I project. The CBCS study is supported by funding from the Capital Region of Denmark. The BFBOCC-LT study was supported by Research Council of Lithuania (SEN-16/2016). The German Consortium for Hereditary Breast and Ovarian Cancer is funded by the German Cancer Aid (110837, 70111850). FJC was supported in part by National Institutes of Health (NIH) grants R01 CA225662, P50 CA116201 and the Breast Cancer Research Foundation. Data for SEARCH was funded by Cancer Research UK (C490/A10119, C490/A10124, C490/A16561 and the Cambridge Cancer Centre) and the US National Institutes of Health (R01CA178535). The University of Cambridge has received salary support in respect of PDPP from the NHS in the East of England through the Clinical Academic Reserve. SAG is a recipient of the Barth Family Chair in Cancer Genetics. HEBCS is funded by Helsinki University Hospital Research Fund, the Sigrid Juselius Foundation and The Cancer Foundation Finland. 

## Notes

The study sponsors had no role in the design of the study; the collection, analysis, and interpretation of the data; the writing of the manuscript; and the decision to submit the manuscript for publication.

ANR has a consultancy arrangement with Abcodia and Everything Genetic Ltd. TVOH has received lecture honoraria from Pfizer. UM has stocks in Abcodia. The other authors have no conflict of interest to declare.

ACA and PDPP conceived and designed the study. XY performed the statistical analysis, ACA supervised the analyses. XY, ACA and PDPP interpreted the results. XY drafted the manuscript; ACA and PDPP reviewed and edited the manuscript. GL collated and harmonized the data from all study centres. All other authors collected, acquired or generated the data. All authors provided critical review of the manuscript and approved the final version. 

This research has been conducted using the UK Biobank Resource under application number 28126. We acknowledge all the families and clinicians who contributed to the participating studies. The FPGMX group acknowledges members of the Cancer Genetics group (IDIS): Ana Blanco, Marta Santamariña, and Belinda Rodríguez-Lage; SWE-BRCA (Swedish BRCA1 and BRCA2 study collaborators): Gothenburg; Sahlgrenska University Hospital: Zakaria Einbeigi and Anna Öfverholm; Linköping University Hospital: Marie Stenmark-Askmalm and Ekaterina Kuchinskaya; Lund University Hospital: Hans Ehrencrona, Therese Törngren, Anders Kvist, and Åke Borg; Stockholm, Karolinska University Hospital: Brita Arver, Annika Lindblom, and Emma Tham; Umeå University Hospital: Beatrice Melin; and Uppsala University Hospital: Ylva Paulsson-Karlsson. The Australian Ovarian Cancer Study Group was supported by the U.S. Army Medical Research and Materiel Command under DAMD17-01-1-0729, The Cancer Council Victoria, Queensland Cancer Fund, The Cancer Council New South Wales, The Cancer Council South Australia, The Cancer Council Tasmania and The Cancer Foundation of Western Australia (Multi-State Applications 191, 211 and 182) and the National Health and Medical Research Council of Australia (NHMRC; ID199600; ID400413 and ID400281). The AOCS gratefully acknowledges additional support from Ovarian Cancer Australia and the Peter MacCallum Foundation. The AOCS also acknowledges the cooperation of the participating institutions in Australia and acknowledges the contribution of the study nurses, research assistants and all clinical and scientific collaborators to the study. The complete AOCS Study Group can be found at www.aocstudy.org. We would like to thank all of the women who participated in these research programs.

## Supplementary Material

djaa030_supplementary_data
