# Supplementary material for: Ovarian and Breast Cancer Risks Associated With Pathogenic Variants in RAD51C and RAD51D
Source: J Natl Cancer Inst. 2020 Feb 28;112(12):1242–50. doi: 10.1093/jnci/djaa030 (PMC7735771; doi:10.1093/jnci/djaa030)
Supplement: djaa030_supplementary_data [file djaa030_supplementary_data.pdf]

Supplementary Material

Methods

Variant frequencies

We estimated the *RAD51C* and *RAD51D* pathogenic variant frequencies in the population using the UK Biobank exome sequencing dataset (http://www.ukbiobank.ac.uk). Specifically, among the 49,960 available subjects, we selected cancer-free individuals (either self-reported or medical records) and removed relatives up to second degree, leaving 42,325 individuals for the variant frequency estimation. The pathogenic variants within *RAD51C* and *RAD51D* were extracted. Variants in the last exon were excluded. The pathogenic variant frequencies were estimated and were used as input parameters in the segregation analysis.

Missing age at cancer diagnosis

Individuals with missing age at cancer diagnosis but other age information available were assumed to develop the corresponding cancer at the minimum available age. For those without any age information available, we assigned the age at cancer diagnosis to be the "average cancer-specific age at diagnosis" obtained from: the family, within the study group and within the country, whichever was available in this order. A summary of the number of individuals with missing age is shown in Supplementary Table 13.

Statistical models

Two main genetic models were fitted: (1) a major-gene model that assumed all familial aggregation of tubo-ovarian carcinoma (TOC) and breast cancer (BC) to be due to *RAD51C* or *RAD51D*; and (2) a polygenic model that considered an additional residual familial component representing other unobserved genetic effects not due to *RAD51C*

or *RAD51D* (1, 2). Under each model, the cancer incidence for individual i at age t born in cohort k from country c was dependent on the underlying genetic effects though a model of the form

$$\lambda_i(t,k,c) = \lambda_0(t,k,c)\,\exp(\beta(t)G_i + P_i),$$

where $\lambda_0(t,k,c)$ is the baseline incidence for non-*RAD51C/D* carriers at age t for cohort k and country c, G_i is an indicator variable taking values 1 for *RAD51C/D* pathogenic variant carriers and 0 for non-carriers, and P_i is the polygenic component which was set to 0 under the single-gene models and was assumed to be normally distributed with mean 0 and variance σ_R^2 under the polygenic models (3, 4). $\beta(t)$ is the log-risk ratio for *RAD51C/D* pathogenic variant relative to non-carriers. To ease interpretation, the models were parameterised in terms of the cancer-specific log-relative risk (log-RR) for *RAD51C* and *RAD51D* pathogenic variant carriers relative to the population incidences for TOC and BC. Specifically, the RR at age t was defined as:

$$\mathrm{RR}\ (\mathrm{t}) = \frac{i_{RAD51C/D+}(t,k,c)}{i_{pop}(t,k,c)}$$

where $\mathrm{i}_{\mathrm{RAD51C/D+}}(\mathrm{t},\mathrm{k},\mathrm{c})$ denotes the average cancer incidence for *RAD51C/D* pathogenic variant carriers at age t born in cohort k from country c (over all polygenic effects) and $\mathrm{i}_{\mathrm{pop}}(\mathrm{t},\mathrm{k},\mathrm{c})$ denotes the population incidence at age t for cohort k and country c.

We constrained the total genetic variance (σ_{total}^2), which was defined as the sum of the variance due to *RAD51C/D* pathogenic variant (σ_K^2) and the residual polygenic variance (σ_R^2), to agree with external estimates of the total polygenic variance. This was assumed be equal to 2.06 for TOC and 1.66 for BC, based on estimates from previously published segregation analyses (1, 5-7).

When the logRR for *RAD51C/D* pathogenic variant carriers relative to the population incidences was assumed to be a piecewise linear function of age, the logRR(t) was modelled as:

$$\mathrm{logRR}((t)) = \begin{cases} a + b_1(t-30), & t \in [30, \tau) \\ a + b_1(\tau - 30) + b_2(t-\tau), & t \in [\tau, 80) \end{cases}$$

where, t is the age, τ is the age-breakpoint where the slope changes to b_2. We optimised τ by fitting a series of models in which τ took values from age 55 to 65 (the plausible age range from the age-specific logRR models).

Cancer incidences

Country- and cohort-specific population cancer incidences (Cancer incidence in five continents, http://ci5.iarc.fr/CI5plus/Default.aspx) were used here to take into account differences in incidences by study group, study location and changes in incidences over time. The overall cancer incidences were constrained over all assumed genetic effects in the model to agree with the population incidences (5). The reported 5-year interval constant incidences were smoothed using the locally weighted regression LOWESS approach (8, 9). A total of eight cohort-specific incidences (<1920, 1920-1929, 1930-1939, 1940-1949, 1950-1959, 1960-1969, 1970-1979 and >1980) were used in the model by assuming each individual was born at the midpoint of each assumed cohort period (1915 for the first cohort and 1985 for the last cohort).

Ascertainment adjustment

We adjusted for ascertainment for each family separately by employing an assumption-free approach (10-12). We divided the data for each family into two parts depending on whether the data could be relevant to the ascertainment (F1) or not (F2). The conditional likelihood L=Pr(F1, F2)/Pr(F1) was then maximized, where Pr(F1, F2) is the probability of the observed data in the entire pedigree and Pr(F1) is the

probability of the observed data in the component relevant to the ascertainment. Specifically, for population-based families, F1 included the phenotype and genotype of the proband only. For families ascertained through multiple affected members, F1 included the genotype of the proband and phenotypes of all the family members. For the families from the four studies that provided data irrespective of the variant screening result (ICR, UKFOCSS, UKFOCR, and SEARCH), the proband's genotype was excluded from F1 as it did not form part of the ascertainment (Supplementary Table 4).

Variant screening sensitivity

Four studies (ICR, UKFOCSS, UKFOCR and SEARCH) provided data on all families screened for *RAD51C* or *RAD51D* variants, irrespective of the mutation search result. Details of these studies and methods have been published elsewhere (13-15). In these families only the proband was screened for *RAD51C/D* mutations. To maximise the number of informative families included in the analysis (after ascertainment adjustment), for these four studies, the analysis included also the families in which the proband was found not to carry a pathogenic variant in *RAD51C* or *RAD51D* and these probands were treated as non-carriers in the analyses. However, this assumes that the variant screening sensitivity, describing the probability of detecting a variant given it exists, is 100%, which may not be necessarily true given the variant screening was carried in research setting in those studies. In practice variant screening sensitivity could be lower and some of the non-carrier families may carry pathogenic variants in *RAD51C* or *RAD51D.* To assess the impact of a reduced variant screening sensitivity on the risk estimates we extended the models to allow for a reduced variant screening sensitivity parameter (16) which was assumed to range from 0.6 to 0.9.

Supplementary Table 1 Previously published studies on tubo-ovarian carcinoma (TOC) risks associated with germline mutations in *RAD51C* and *RAD51D*

**Published case-control studies**							
**Population/ country**	**Samples**		**Minor allele frequency**		**OR (95% CI)**		**Reference**
	**Cases**	**Controls**	***RAD51C***	***RAD51D***	***RAD51C***	***RAD51D***	
European	~120,000 BC*/TOC†	~120,000	NA	NA	4.24 (2.56-7.02)	7.28 (4.03-13.14)	(17)
France	5131 patients with FH‡ of BC or TOC	571 geographically matched controls	0.0012	0.00052	14.62 (5.39-29.52)	11.84 (1.09-40.00)	(18)
United States	1,915 patients unselected for FH	4,300 ESP§ European American	0.0002	0.0005	15.8 (1.9-128)	9.0 (1.9-42.5)	(19)
		3,6276 ExAC	0.0011	0.0004	3.4 (1.5-7.6)	10.9 (4.6-26.0)	
Mixed population	3.429 patients (including 3,135 unselected for FH and 294 with FH)	2,772 controls (including 2,678 unselected for FH and 94 selected for FH)	0.00036	0.00018	5.2 (1.1-24)	12 (1.5-90)	(15)
**Published family segregation studies**							
**Population/ country**	**Families**		**Minor allele frequency**		**HR (95% CI)**		**Reference**
			RAD51C	***RAD51D***	***RAD51C***	***RAD51D***	
European	1132 families with FH		NA	NA	5.88 (2.91-11.88)	NA	(14)
UK	911 families with FH of BC/TOC		NA	NA	NA	6.30 (2.86-13.85)	(13)

*BC: breast cancer

†TOC: tubo-ovarian carcinoma

‡FH: family history

§ESP: the National, Heart, Lung, and Blood Institute Exome Sequencing Project

Supplementary Table 2 Previously published studies on breast cancer risks associated with germline mutations in *RAD51C* and *RAD51D*

**Published case-control studies**							
**Population/ country**	**Samples**		**Minor allele frequency**		**OR (95% CI)**		**Reference**
	**Cases**	**Controls**	***RAD51C***	***RAD51D***	***RAD51C***	***RAD51D***	
Australia	3080 patients with FH* of BC† or TOC‡	4840 geographocally matched controls	0.0004	NA	8.67 (1.89-80.52)	NA	(20)
European	~120,000 BC/TOC	~120,000	NA	NA	1.13 (0.88-1.44)	1.25 (0.90-1.75)	(17)
France	5131 patients with FH of BC or TOC	571 geographically matched controls	0.0012	0.00052	1.92 (0.71-3.85)	2.42 (0.36-7.39)	(18)
Germany	5,589 Patients with FH or early-onset BC or bilateral BC or patients affected by BC and TOC	2,189 geographically matched controls	0.00045	0	1.76 (0.38-8.17)	NA	(21)
		27,173 ExAC (European, non-Finnish, non-TCGA)	0.00065	0.00015	1.29 (0.62-2.69)	3.04 (0.99-9.30)	
		7,325 FLOSSIES (European American ancestry)	0.00015	0.00015	5.91 (1.28-27.34)	3.28 (0.64-16.91)	
United States (white or Ashkenazi Jewish)	38,326 patients quantifying for clinical genetic testing	26,911 ExAC (non-Finnish, non-TCGA)	0.0006	0.0001	0.78 (0.47-1.37)	3.07 (1.21-7.88)	(22)
Mixed population	2,134 patients with FH of BC or TOC	26,375 ExAC (non-Finnish, non-TCGA European)	0.0007	0.0001	0.39 (0.02-2.41)	8.33 (2.20-30.48)	(23)
**Published family segregation studies**							
**Population/ country**	**Families**		**Minor allele frequency**		**HR (95% CI)**		**Reference**
			RAD51C	***RAD51D***	***RAD51C***	***RAD51D***	
European	1132 families with FH		NA	NA	0.91 (0.45-1.86)	NA	(14)
UK	911 families with FH of BC/TOC		NA	NA	NA	1.32 (0.59-2.96)	(13)

*FH: family history

†BC: breast cancer

‡TOC: tubo-ovarian carcinoma

Supplementary Table 3 List of contributing study groups and number of families

Study group	Full name of study groups	Total number of families		Number of families by ascertainment type		Number of non-informative families excluded from the analysis due to ascertainment		Number of families eligible for inclusion in the analysis with pathogenic variants‡		Reference
		RAD51C	***RAD51D***	**fhx***	**pop†**	***RAD51C***	***RAD51D***	***RAD51C***	***RAD51D***	
Ambry	Ambry Genetics	18	10	28	0	7	5	11	5	
AOCS	Australian Ovarian Cancer Study	3	1	0	4	0	0	3	1	
BFBOCC-LT	Baltic Familial Breast Ovarian Cancer Consortium (Lithuania)	4	0	4	0	2	0	2	0	
CBCS	Copenhagen Breast Cancer Study	7	1	8	0	3	1	4	0	
CFB		15	5	20	0	13	5	2	0	
CNIO	Spanish National Cancer Centre	1	0	1	0	1	0	0	0	
Curie	Institut Curie	1	3	4	0	0	3	1	0	
DFCI	Dana Farber Cancer Insitute	4	2	6	0	3	2	1	0	
FPGMX	Fundación Pública Galega de Medicina Xenómica	0	1	1	0	0	0	0	1	
GC-HBOC	German Consortium for Hereditary Breast and Ovarian Cancer	74	16	90	0	26	8	48	8	

**Study group**	**Full name of study groups**	**Total number of families**		**Number of families by ascertainment type**		**Number of non-informative families excluded from the analysis due to ascertainment**		**Number of families eligible for inclusion in the analysis with pathogenic variants‡**		**Reference**
		RAD51C	***RAD51D***	**fhx***	**pop†**	***RAD51C***	***RAD51D***	***RAD51C***	***RAD51D***	
HCSC	Hospital Clinico San Carlos	1	1	2	0	0	1	1	0	
HEBCS	Helsinki Breast Cancer Study	6	4	8	2	2	1	4	3	
HVH	University Hospital Vall d'Hebron	0	3	3	0	0	1	0	2	(24)
IBOC		1	0	1	0	0	0	1	0	
ICR	BOCS (Breast and Ovarian Cancer Study) formerly FBCS (Familial Breast Cancer Study	5354 (among these, 4451 families were screened for *RAD51C* and 5026 families were screened for *RAD51D*)		5354	0	0	0	4451 among these 24 with pathogenic variants	5026 among these 21 with pathogenic variants	(13, 14) Sequencing methods described in study references
kConFab	Kathleen Cuningham Consortium for Research into Familial Breast Cancer	2	1	3	0	0	0	2	1	
MALOVA	MALignant OVArian cancer study	1	2	0	3	0	0	1	2	(25)
MCBCS		1	0	1	0	1	0	0	0	
MCGILL	McGill University	1	1	2	0	1	0	0	1	(26)
MSKCC	Memorial Sloane Kettering Cancer Center	1	0	1	0	0	0	1	0	
POC		3	0	3	0	3	0	0	0	

Study group	Full name of study groups	Total number of families		Number of families by ascertainment type		Number of non-informative families excluded from the analysis due to ascertainment		Number of families eligible for inclusion in the analysis with pathogenic variants‡		Reference
		RAD51C	*RAD51D*	fhx*	pop†	*RAD51C*	*RAD51D*	*RAD51C*	*RAD51D*	
UKFOCSS/ UKFOCR	UK Familial Ovarian Cancer Screening Study/ UK Familial Ovarian Cancer Registry	491 (among these, 486 families were screened for *RAD51C* and 484 families were screened for *RAD51D*)		491	0	0	0	486 among these 8 with pathogenic variants	484 among these 6 with pathogenic variants	(27) Sequencing methods described in reference (15)
SEARCH		1158 (among these, 1151 families were screened for *RAD51C* and 1154 families were screened for *RAD51D*)		0	1158	0	0	1151 among these 3 with pathogenic variants	1154 among these 7 with pathogenic variants	(15) Sequencing methods described in study reference.
SWE-BRCA	Swedish Breast Cancer Study	9	1	10	0	3	0	6	1	
UCV		0	2	2	0	0	2	0	0	
UPENN	University of Pennsylvania	1	0	1	0	1	0	0	0	
USC	University of South California	2	2	4	0	0	1	2	1	
Total		6244	6720	6049	1167	66	30	6178 among these 125 with pathogenic variants	6690 among these 60 with pathogenic variants	

*fhx: family-based ascertainment

†pop: population-based ascertainment

‡For ICR, SEARCH and UKFOCSS/UKFOCR the cell contains the total number of families screened for *RAD51C* or *RAD51D*

Supplementary Table 4 Summary of types of ascertainment adjustment schemes used in the study

Type of ascertainment	Study Groups	F1: Data relevant to ascertainment	F2: Data not relevant to ascertainment
Population-based	SEARCH	(1) Phenotype of the proband	(1) Phenotypes of all family members except the proband; (2) mutation status of all family members
	Others	(1) Phenotype of the proband; (2) mutation status of the proband	(1) Phenotypes of all family members except the proband; (2) mutation status of all family members except proband's
family-based	ICR, UKFOCSS, UKFOCR	(1) All family phenotypes	Mutation status of all family members
	Others	(1) All family phenotypes; (2) mutation status of the proband	Mutation status of all family members except proband's

Supplementary Table 5 List of pathogenic variants in *RAD51C* among eligible families included in the analysis

Variants HGVS (ref: ENST00000337432.9)	Type	Number of families
c.158_160delinsTT	frameshift variant	1
c.158del	frameshift variant	1
c.181_182del	frameshift variant	2
c.186_187del	frameshift variant	1
c.216_220del	frameshift variant	2
c.224dup	frameshift variant	6
c.483_484insC	frameshift variant	2
c.498del	frameshift variant	2
c.501_502dup	frameshift variant	1
c.525dup	frameshift variant	3
c.622_623del	frameshift variant	1
c.651_652del	frameshift variant	1
c.704dup	frameshift variant	1
c.732del	frameshift variant	4
c.774del	frameshift variant	3
c.849_852del	frameshift variant	1
c.862del	frameshift variant	3
c.890del	frameshift variant	1
c.93del	frameshift variant	14
c.945dup	frameshift variant	1
c.966-?_c.1131+?del	frameshift variant	1
c.572-?_c.1131+?del	frameshift variant	1
c.706-?_c.1131+?del	frameshift variant	12
c.966-?_c.1026+?del	frameshift variant	2
c.706-?_c.837+?del	in-frame large deletion	1
c.145+1G>T	intron splicing site variant	2
c.146-4_146-2del	intron splicing site variant	1
c.404+2T>C	intron splicing site variant	2
c.571+1G>A	intron splicing site variant	2
c.572-1G>T	intron splicing site variant	1
c.705+1G>A	intron splicing site variant	1
c.706-1G>A	intron splicing site variant	3
c.706-2A>G	intron splicing site variant	14
c.837+1G>A	intron splicing site variant	2
c.905-2del	intron splicing site variant	3
c.397C>T	nonsense variant	3
c.502A>T	nonsense variant	2
c.577C>T	nonsense variant	6
c.664C>T	nonsense variant	1
c.701C>G	nonsense variant	2
c.955C>T	nonsense variant	7
c.97C>T	nonsense variant	4
c.994C>T	nonsense variant	1

Supplementary Table 6 List of pathogenic variants in *RAD51D* among eligible families included in the analysis

**Variants HGVS (ref: ENST00000345365.10)**	**Type**	**Number of families**
c.140_141insAA	frameshift variant	1
c.255_256insCTCCCAAAGTGCTAGG	frameshift variant	1
c.270_271dup	frameshift variant	1
c.363del	frameshift variant	2
c.416del	frameshift variant	1
c.480+1G>A	frameshift variant	1
c.564_567del	frameshift variant	2
c.564del	frameshift variant	2
c.623dup	frameshift variant	1
c.667_667+21del	frameshift variant	1
c.740_741dup	frameshift variant	1
c.748del	frameshift variant	5
c.83-?_577-?del	frameshift variant	1
c.145-?_263+?del	frameshift variant	1
c.451C>T	nonsense variant	1
c.478C>T	nonsense variant	1
c.547C>T	nonsense variant	1
c.556C>T	nonsense variant	11
c.620C>A	nonsense variant	1
c.649G>T; c.655C>T (cis)	nonsense variant	1
c.694C>T	nonsense variant	4
c.757C>T	nonsense variant	2
c.803G>A	nonsense variant	3
c.898C>T	nonsense variant	4
c.263+1G>A	intron splicing site variant	1
c.576+1G>A	intron splicing site variant	5
c.577-2A>G	intron splicing site variant	2
c.649_655delinsTGAGGTT	intron splicing site variant	1
c.83-1G>A	intron splicing site variant	1

Supplementary Table 7 Estimated age-specific cancer incidences and cumulative cancer risks for *RAD51C* and *RAD51D* pathogenic variant carriers in the USA.

Age (years)	Estimated incidences (per 1,000 person-years) for *RAD51C* and *RAD51D* pathogenic variant carriers (95% Confidence Interval)*			
	RAD51C		***RAD51D***	
	**BC**	**TOC**	**BC**	**TOC**
30	0.4 (0.3-0.6)	0.06 (0.02-0.2)	0.4 (0.3-0.6)	0.04 (0.009-0.2)
40	2 (1-3)	0.3 (0.1-0.7)	2 (1-3)	0.2 (0.1-0.6)
50	4 (3-6)	1 (1-2)	4 (3-6)	1 (0.9-2)
60	7 (5-9)	5 (3-8)	6 (4-9)	4 (3-6)
70	9 (6-13)	2 (0.9-6)	8 (6-12)	3 (2-7)
79	9 (6-13)	0.9 (0.1-6)	8 (6-12)	2 (0.6-9)
**Age (years)**	**Estimated cumulative risks (%) for *RAD51C* and *RAD51D* pathogenic variant carriers by age (95% Confidence Interval)***			
	RAD51C		***RAD51D***	
	**BC**	**TOC**	**BC**	**TOC**
30	0.1 (0.1-0.2)	0.04 (0.04-0.04)	0.1 (0.09-0.2)	0.04 (0.04-0.04)
40	1 (0.8-2)	0.2 (0.09-0.4)	1 (0.7-2)	0.1 (0.07-0.4)
50	4 (3-6)	0.9 (0.5-2)	4 (3-6)	0.8 (0.4-1)
60	9 (6-13)	4 (2-6)	8 (6-12)	3 (2-6)
70	16 (11-22)	7 (4-11)	15 (10-21)	7 (5-11)
80	23 (17-31)	8 (5-17)	21 (15-30)	10 (6-18)

*Assuming the USA population calendar and cohort specific incidences for an individual born between 1950-1959. Mortality is not accounted for absolute risk estimate

BC: breast cancer; TOC: tubo-ovarian carcinoma

Supplementary Table 8 Estimated relative risks (RRs) of tubo-ovarian carcinoma (TOC) and breast cancer (BC) for *RAD51C* and *RAD51D* pathogenic variant carriers by birth cohort

Cancer	Year of birth	***RAD51C***		***RAD51D***	
		**RR (95% CI)**	**p-value***	**RR (95% CI)**	**p-value***
BC	Before 1940	1	0.15	1	0.57
	1940-1959	2.47 (0.77-7.93)		1.43 (0.5-4.09)	
	in 1960 or later	2.68 (0.81-8.84)		1.82 (0.57-5.81)	
TOC	Before 1940	1	0.43	1	0.75
	1940-1959	1.19 (0.54-2.62)		1.17 (0.53-2.61)	
	in 1960 or later	0.53 (0.13-2.16)		0.76 (0.23-2.56)	

*Likelihood ratio test comparing against the model with a constant RR, degrees of freedom=2

Supplementary Table 9 Estimated breast cancer (BC) and tubo-ovarian carcinoma (TOC) relative risks for *RAD51C* and *RAD51D* pathogenic variant carriers by different variant screening sensitivity parameters*

**Gene**	**Cancer**	**Assumed sensitivity of mutation screening**			
		**0.9**	**0.8**	**0.7**	**0.6**
RAD51C	BC	2.08 (1.46-2.98)	2.16 (1.51-3.10)	2.25 (1.57-3.24)	2.37 (1.64-3.43)
	TOC	8.29 (6.07-11.33)	8.94 (6.45-12.37)	9.75 (6.93-13.71)	10.86 (7.58-15.56)
RAD51D	BC	1.90 (1.28-2.82)	1.98 (1.33-2.94)	2.06 (1.38-3.07)	2.15 (1.44-3.22)
	TOC	8.22 (5.98-11.29)	8.86 (6.35-12.35)	9.72 (6.87-13.75)	10.89 (7.56-15.70)

*Under the models assuming a constant RR across age groups.

Supplementary Table 10 Age-specific cumulative breast cancer (BC) risks (%) for female *RAD51C* and *RAD51D* pathogenic variant carriers by cancer family history

Age (years)	Without considering family history	Mother unaffected at 50, maternal grandmother unaffected at 70	Mother with BC at 35	Mother and sister with BC at 50	Mother and maternal grandmother with BC at 50
RAD51C					
30	0.1 (0.1-0.2)	0.1 (0.1-0.2)	0.2 (0.2-0.3)	0.3 (0.2-0.5)	0.2 (0.2-0.3)
35	0.4 (0.3-0.6)	0.4 (0.3-0.5)	0.7 (0.5-1)	1 (0.8-2)	0.8 (0.6-1)
40	1 (0.7-1)	1 (0.7-1)	2 (1-3)	3 (2-4)	2 (2-3)
45	2 (2-3)	2 (2-3)	4 (3-6)	6 (4-8)	5 (3-6)
50	4 (3-6)	4 (3-5)	7 (5-10)	11 (8-14)	8 (6-11)
55	6 (4-9)	6 (4-9)	11 (8-16)	16 (12-22)	13 (9-17)
60	9 (6-13)	9 (6-12)	16 (11-22)	23 (17-30)	18 (13-24)
65	12 (9-17)	12 (8-16)	21 (15-28)	29 (22-38)	23 (17-31)
70	15 (11-21)	15 (11-20)	26 (19-34)	36 (27-45)	29 (21-37)
75	18 (13-25)	18 (13-24)	30 (22-39)	41 (32-51)	33 (25-43)
80	21 (15-29)	21 (15-28)	34 (26-45)	46 (36-57)	38 (29-48)
RAD51D					
30	0.1 (0.1-0.2)	0.1 (0.1-0.2)	0.2 (0.1-0.3)	0.3 (0.2-0.4)	0.2 (0.2-0.3)
35	0.4 (0.2-0.5)	0.4 (0.2-0.5)	0.7 (0.5-1)	1 (0.7-2)	0.8 (0.5-1)
40	0.9 (0.6-1)	0.9 (0.6-1)	2 (1-3)	3 (2-4)	2 (1-3)
45	2 (1-3)	2 (1-3)	4 (3-5)	6 (4-8)	4 (3-6)
50	4 (3-5)	4 (2-5)	7 (5-10)	10 (7-14)	8 (5-11)
55	6 (4-9)	6 (4-8)	10 (7-15)	15 (11-21)	12 (8-17)
60	8 (6-12)	8 (6-12)	15 (10-21)	21 (15-29)	16 (12-23)
65	11 (8-16)	11 (7-15)	19 (14-27)	27 (20-36)	22 (15-30)
70	14 (10-20)	14 (9-19)	24 (17-33)	33 (25-44)	27 (19-36)
75	17 (12-24)	16 (11-23)	28 (20-38)	39 (29-50)	31 (23-41)
80	20 (14-28)	19 (13-27)	32 (23-43)	44 (33-55)	36 (26-47)

Supplementary Table 11 Age-specific cumulative tubo-ovarian carcinoma (TOC) risks (%) for female *RAD51C* and *RAD51D* pathogenic variant carriers by cancer family history

Age (years)	Without considering family history	Mother unaffected at 50, maternal grandmother unaffected at 70	Mother with TOC at 55	Mother and sister with TOC at 50	Mother and maternal grandmother with TOC at 50
RAD51C					
35	0.1 (0-0.2)	0.1 (0-0.1)	0.1 (0.1-0.3)	0.2 (0.1-0.5)	0.2 (0.1-0.3)
40	0.2 (0.1-0.4)	0.2 (0.1-0.4)	0.3 (0.2-0.8)	0.6 (0.3-1)	0.4 (0.2-0.9)
45	0.4 (0.2-0.9)	0.4 (0.2-0.9)	0.8 (0.4-2)	2 (0.7-3)	1 (0.5-2)
50	1 (0.6-2)	1 (0.6-2)	2 (1-4)	4 (2-6)	2 (1-4)
55	2 (1-4)	2 (1-3)	4 (3-7)	7 (5-11)	5 (3-8)
60	4 (3-7)	4 (3-7)	9 (6-12)	14 (10-20)	10 (7-15)
65	7 (5-11)	7 (5-11)	14 (9-20)	22 (16-31)	16 (11-23)
70	9 (6-15)	9 (6-14)	17 (11-25)	27 (19-38)	20 (13-29)
75	10 (6-18)	10 (6-18)	19 (12-30)	30 (20-45)	22 (14-35)
80	11 (6-21)	11 (6-21)	20 (12-35)	32 (20-51)	24 (14-40)
RAD51D					
35	0 (0-0.1)	0 (0-0.1)	0.1 (0.1-0.2)	0.2 (0.1-0.4)	0.1 (0.1-0.3)
40	0.1 (0.1-0.3)	0.1 (0.1-0.3)	0.2 (0.1-0.6)	0.4 (0.2-1)	0.3 (0.1-0.8)
45	0.3 (0.2-0.8)	0.3 (0.2-0.8)	0.6 (0.3-2)	1 (0.5-3)	0.8 (0.4-2)
50	0.8 (0.5-2)	0.8 (0.5-2)	2 (0.9-3)	3 (2-5)	2 (1-4)
55	2 (1-3)	2 (1-3)	4 (3-6)	7 (4-10)	5 (3-7)
60	4 (3-7)	4 (3-7)	8 (6-12)	14 (9-20)	10 (7-15)
65	7 (5-11)	7 (5-10)	13 (9-19)	22 (15-30)	16 (11-22)
70	9 (6-14)	9 (6-14)	17 (12-25)	28 (19-38)	20 (14-29)
75	11 (7-19)	11 (7-18)	20 (13-31)	32 (23-46)	24 (16-36)
80	13 (7-23)	13 (7-23)	23 (14-37)	36 (23-54)	27 (17-43)

Supplementary Table 12 Estimated tubo-ovarian carcinoma (TOC) and breast cancer (BC) RR for *RAD51C* and *RAD51D* pathogenic variant carriers under the best fitting models in the main text assuming censoring for risk-reducing surgery occurs one year after surgery for both affected and unaffected*.

Cancer	Models considered	Age (years)	*RAD51C* RR (95% CI)	AIC
RAD51C				
TOC	Piecewise linear model†	35	2.40	4328.6
		45	5.14	
		55	11.02	
		65	9.01	
		75	2.81	
BC	Age-constant model	20-79	1.99 (1.39-2.85)	4346.5
RAD51D				
TOC	Piecewise linear model‡	35	1.64	4151.7
		45	4.30	
		55	11.29	
		65	10.14	
		75	5.75	
BC	Age-constant model	20-79	1.83 (1.24-2.72)	4178.0

*There was only 1 unaffected woman in families with *RAD51D* pathogenic variants censored at risk-reducing bilateral mastectomy. The number of unaffected women who had undergone risk-reducing salpingo-oophorectomy were: 8 among the families with *RAD51C* pathogenic variants, and 5 among the families with *RAD51D* pathogenic variants.

†$\log RR(t)=a+b_1(t-30)$ if $t \in [30,60)$; $\log RR(t)=a+b_1\times 30+b_2(t-60)$ if $t \in [60,80)$ where $a=0.49$ (95% CI: -0.75 to 1.74), $b_1=0.076$ (95% CI: 0.025 to 0.13), $b_2=-0.12$ (95% CI: -0.23 to -0.0043)

‡$\log RR(t)=a+b_1(t-30)$ if $t \in [30,58)$; $\log RR(t)=a+b_1\times 28+b_2(t-58)$ if $t \in [58,80)$ where $a=0.011$ (95% CI: -1.52 to 1.55), $b_1=0.097$ (95% CI: 0.033 to 0.16), $b_2=-0.057$ (95% CI: -0.13 to 0.016)

Supplementary Table 13 A summary of the number of individuals with missing age information at different events (based on all families used in the analysis).

Event	Total number of individuals with event	Total number of individuals with missing ages at event (%)	Number of individuals with missing ages at each event and age inferred from:		
			other age information on the individual (%)	the mean event age within the family (%)	the mean event age within the study group (%)
First breast cancer (female)	15850	2378 (15%)	1426 (9%)	871 (5.5%)	81 (0.5%)
Ovarian cancer	6742	920 (13.65%)	657 (9.7%)	166 (2.5%)	97 (1.4%)
First other cancer (female)	6172	1551 (25.13%)	1014 (16.4%)	277 (4.5%)	260 (4.2%)
Bilateral mastectomy	144	29 (20.14%)	29 (20.1%)	—	—
Bilateral salpingo-oophorectomy	624	42 (6.73%)	42 (6.7%)	—	—

References

1. Antoniou AC, Casadei S, Heikkinen T, et al. Breast-Cancer Risk in Families with Mutations in PALB2. New England Journal of Medicine. 2014;371(6):497-506.
2. Yang X, Leslie G, Doroszuk A, et al. Cancer risks associated with gernline PALB2 pathogenic variants - an international study of 524 families. Journal of Clinical Oncology.(in press).
3. Antoniou AC, Pharoah PDP, McMullan G, et al. A comprehensive model for familial breast cancer incorporating BRCA1, BRCA2 and other genes. British journal of cancer. 2002;86(1):76-83.
4. Lange K. An approximate model of polygenic inheritance. Genetics. 1997;147(3):1423-30.
5. Antoniou AC, Pharoah PD, McMullan G, Day NE, Ponder BA, Easton D. Evidence for further breast cancer susceptibility genes in addition to BRCA1 and BRCA2 in a population-based study. Genet Epidemiol. 2001;21(1):1-18.
6. Jervis S, Song H, Lee A, et al. A risk prediction algorithm for ovarian cancer incorporating BRCA1, BRCA2, common alleles and other familial effects. J Med Genet. 2015;52(7):465-75.
7. Pharoah PDP, Antoniou A, Bobrow M, Zimmern RL, Easton DF, Ponder BAJ. Polygenic susceptibility to breast cancer and implications for prevention. 2002.
8. Cleveland WS. Robust locally weighted regression and smoothing scatterplots. Journal of the American Statistical Association. 1979;74:829-36.
9. Lee AJ, Cunningham AP, Kuchenbaecker KB, Mavaddat N, Easton DF, Antoniou AC. BOADICEA breast cancer risk prediction model: updates to cancer incidences, tumour pathology and web interface. British Journal of Cancer. 2014;110(2):535-45.
10. Cannings C, Thompson EA. Ascertainment in the sequential sampling of pedigrees. Clin Genet. 1977;12(4):208-12.
11. Ewens WJ, Shute NC. A resolution of the ascertainment sampling problem. I. Theory. Theoretical population biology. 1986;30:388-412.
12. Shute NC, Ewens WJ. A resolution of the ascertainment sampling problem. III. Pedigrees. Am J Hum Genet. 1988;43:387-95.
13. Loveday C, Turnbull C, Ramsay E, et al. Germline mutations in RAD51D confer susceptibility to ovarian cancer. Nat Genet. 2011;43(9):879-82.
14. Loveday C, Turnbull C, Ruark E, et al. Germline RAD51C mutations confer susceptibility to ovarian cancer. Nat Genet. 2012;44(5):475-6; author reply 6.
15. Song H, Dicks E, Ramus SJ, et al. Contribution of Germline Mutations in the RAD51B, RAD51C, and RAD51D Genes to Ovarian Cancer in the Population. J Clin Oncol. 2015;33(26):2901-7.
16. Antoniou AC, Cunningham AP, Peto J, et al. The BOADICEA model of genetic susceptibility to breast and ovarian cancers: updates and extensions. Br J Cancer. 2008;98(8):1457-66.
17. Suszynska M, Klonowska K, Jasinska AJ, Kozlowski P. Large-scale meta-analysis of mutations identified in panels of breast/ovarian cancer-related genes - Providing evidence of cancer predisposition genes. Gynecol Oncol. 2019;153(2):452-62.
18. Castera L, Harter V, Muller E, et al. Landscape of pathogenic variations in a panel of 34 genes and cancer risk estimation from 5131 HBOC families. Genet Med. 2018;20(12):1677-86.
19. Norquist BM, Harrell MI, Brady MF, et al. Inherited Mutations in Women With Ovarian Carcinoma. JAMA Oncol. 2016;2(4):482-90.
20. Li N, McInerny S, Zethoven M, et al. Combined tumor sequencing and case/control analyses of RAD51C in breast cancer. J Natl Cancer Inst. 2019.
21. Hauke J, Horvath J, Gross E, et al. Gene panel testing of 5589 BRCA1/2-negative index patients with breast cancer in a routine diagnostic setting: results of the German Consortium for Hereditary Breast and Ovarian Cancer. Cancer Med. 2018;7(4):1349-58.
22. Couch FJ, Shimelis H, Hu C, et al. Associations Between Cancer Predisposition Testing Panel Genes and Breast Cancer. JAMA Oncol. 2017;3(9):1190-6.
23. Slavin TP, Maxwell KN, Lilyquist J, et al. The contribution of pathogenic variants in breast cancer susceptibility genes to familial breast cancer risk. NPJ Breast Cancer. 2017;3:22.

24. Feliubadalo L, Lopez-Fernandez A, Pineda M, et al. Opportunistic testing of BRCA1, BRCA2 and mismatch repair genes improves the yield of phenotype driven hereditary cancer gene panels. Int J Cancer. 2019;145(10):2682-91.
25. Glud E, Kjaer SK, Thomsen BL, et al. Hormone therapy and the impact of estrogen intake on the risk of ovarian cancer. Arch Intern Med. 2004;164(20):2253-9.
26. Rivera B, Di Iorio M, Frankum J, et al. Functionally Null RAD51D Missense Mutation Associates Strongly with Ovarian Carcinoma. Cancer Res. 2017;77(16):4517-29.
27. Rosenthal AN, Fraser LSM, Philpott S, et al. Evidence of Stage Shift in Women Diagnosed With Ovarian Cancer During Phase II of the United Kingdom Familial Ovarian Cancer Screening Study. J Clin Oncol. 2017;35(13):1411-20.
